# Horizontal transfer of RNA and proteins between cells by extracellular microvesicles: 14 years later

**DOI:** 10.1186/s40169-016-0087-4

**Published:** 2016-03-04

**Authors:** Mariusz Z. Ratajczak, Janina Ratajczak

**Affiliations:** Stem Cell Institute at James Graham Brown Cancer Center, University of Louisville, 500 S. Floyd Street, Rm. 107, Louisville, KY 40202 USA; Department of Regenerative Medicine, Medical University of Warsaw, Warsaw, Poland

**Keywords:** RNA, ExMVs, Horizontal transfer of RNA, Exosomes, Regenerative medicine, Circulating RNA, Liquid biopsies

## Abstract

Extracellular microvesicles (ExMVs) are part of the cell secretome, and evidence has accumulated for their involvement in several biological processes. Fourteen years ago our team demonstrated for the first time that ExMVs carry functional RNA species and proteins from one cell to another, an observation that opened up the new research field of horizontal transfer of bioactive molecules in cell-to-cell communication. Moreover, the presence of mRNA, noncoding RNA, and miRNA in ExMVs in blood and other biological fluids opened up the possibility of employing ExMVs as new detection markers for pathological processes, and ExMVs became a target for “liquid biopsy” approaches. While ExMV-derived mRNAs may be translated in target cells into appropriate proteins, miRNAs regulate expression of corresponding mRNA species, and both RNA-depended ExMV-mediated mechanisms lead to functional changes in the target cells. Following from this observation, several excellent papers have been published that confirm the existence of the horizontal transfer of RNA. Moreover, in addition to RNA, proteins, bioactive lipids, infectious particles and intact organelles such as mitochondria may follow a similar mechanism. In this review we will summarize the impressive progress in this field—14 years after initial report.

## Introduction

**Fig. 1:**
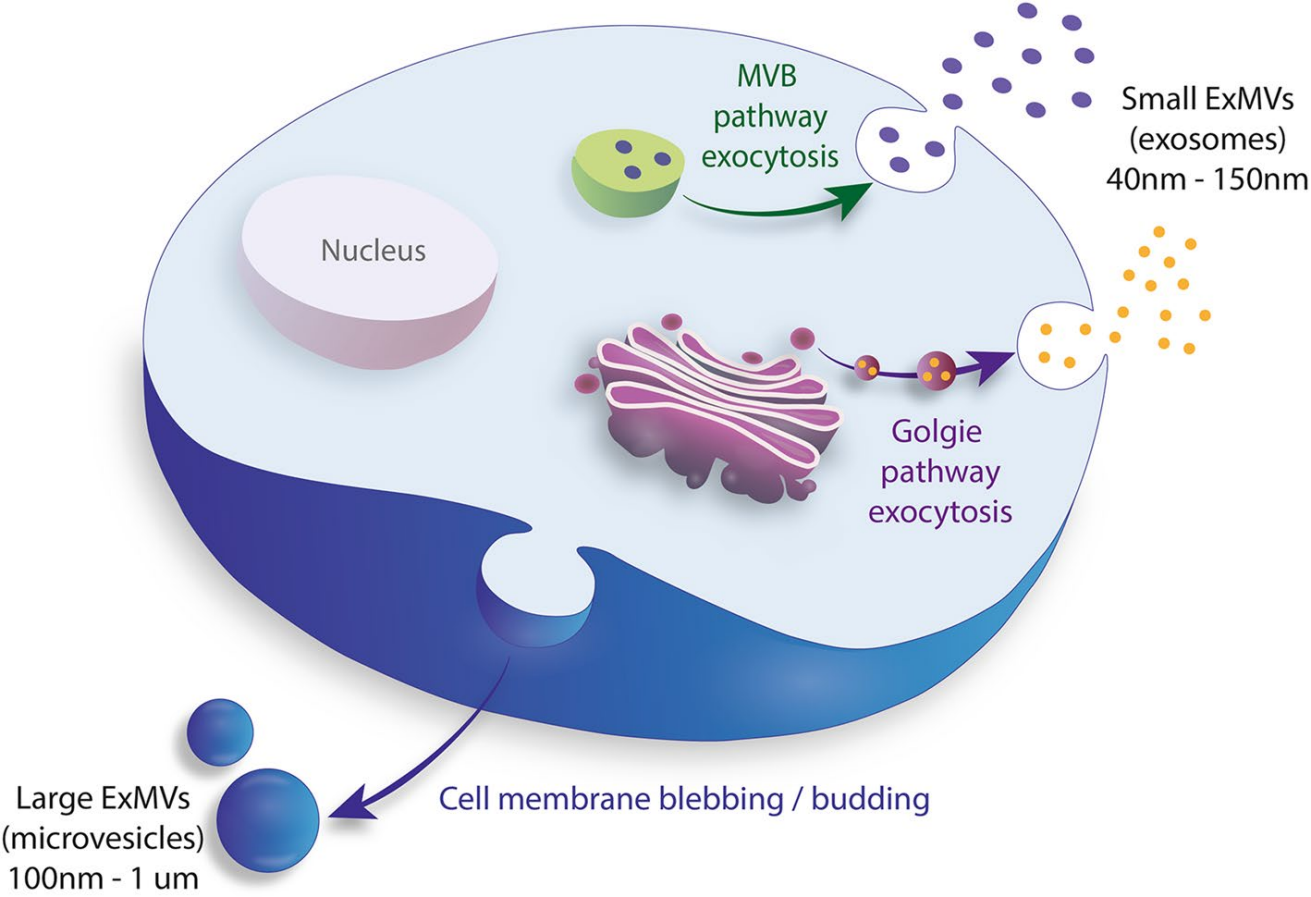
Upon activation, every cell type secretes ExMVs. Larger ExMVs (microvesicles) are released from the cell surface by blebbing and budding of the cell membrane, Smaller ExMVs (exosomes) are initiated in endosomes as intraluminal vesicles in multivesicular bodies (MVBs) after endocytosis of pathogens or due to activation of cells by other stimuli, or are generated in the Golgi apparatus during secretion of cell-synthesized proteins

Both single-celled organisms (e.g., bacteria, protozoea) and cells that are part of multicellular organisms communicate with the environment and other cells by several mechanisms. The best known and studied so far are ligand–receptor-based interactions that involve peptides, bioactive lipids, extracellular nucleotides, and the corresponding specific receptors on the cell surface or in the cell cytoplasm that bind these ligands. Interestingly, evidence has accumulated that the one of most developmentally early cell-to-cell communication mechanism involves spherical membrane fragments shed from the cell surface or the endosomal compartment, which have been described collectively as microparticles, microvesicles, or exosomes [1–5]. This communication mechanism is preserved in all species, and small spherical membrane fragments are currently called extracellular microvesicles (ExMVs), as recommended by the International Society for Extracellular Vesicles [2]. While larger ExMVs (~100 nm–1 μm in diameter) are shed from lipid raft-enriched cell surface membrane domains by blebbing and budding of the cell membrane, smaller ExMVs (~40–150 nm), also known as exosomes, are derived from the endosomal cell membrane compartment and originate from multivesicular bodies (MVBs) or from the release of Golgi apparatus-derived vesicles in the process of exocytosis (Fig. 1) [1–6]. Whatever their source, ExMVs that are released from normal healthy cells should be distinguished from apoptotic bodies that originate in dying cells. It is important to keep in mind this difference, because some small apoptotic bodies could be co-isolated with ExMVs [2].

The fact that ExMVS are present in biological fluids or in conditioned media harvested from cells cultured in vitro has been known for many years, and it has been suggested step by step by some investigators that these small spherical membrane structures play an important role in several biological processes. For example, peripheral blood platelet-derived ExMVs have been demonstrated to be involved in the coagulation process [7], mesenchymal stromal cell (MSC)-derived ExMVs in bone mineralization [8], and B cell-derived ExMVs in regulation of certain T cell-mediated immune responses [9]. Nevertheless, for many years there was skepticism about the role of these membrane fragments in regulating cells, and they were often dismissed as “cell debris” released from damaged cells. Thus, many of the biological effects of ExMVs were considered to be artifacts, and it took some time to convince the scientific community that ExMVs may be released from normal healthy cells. Now it seems likely that the trafficking of ExMVs was one of the first cell-to-cell communication mechanisms that emerged during evolution and anticipated the development of more specific ligand-receptor interactions [1–6].

Following on this concept, some papers have been published showing that ExMVs act as signaling device and activate target cells by ligands expressed on the ExMV surface [10, 11] or by the transfer of membrane receptors from one cell to another [12]. However, one of the major problems with moving this field forward has been the lack of established methods to isolate, measure the concentration of, and purify ExMVs from biological fluids. Some of these problems remain today, and several approaches have been proposed to unify isolation and enumeration protocols [2].

The most likely explanation for the rapid development of ExMV research, which has been followed by an exponential increase in the number of published papers in subsequent years, has been the demonstration that these small cellular membrane fragments transfer RNA species and several other biologically active molecules between cells and thus may induce functional changes in the target cells [13–15]. These observations became particularly important at the time of discovery of stem cell plasticity, when some of the markers derived from cells employed as therapeutics were detected in cells in the damaged tissues. Rather than as a fusion phenomenon, this phenotypic and functional modification of target cells in damaged organs could at least partially be explained by the transfer of bioactive molecules from the cells employed as therapeutics [1, 16]. Moreover, the fact that ExMVs often possess a unique molecular signature that depends on the cell of origin, providing a kind of “business card” with which to identify these cells, has opened the door to exploring their application as diagnostic tools to identify pathologic changes in the body. Based on this approach, ExMVs emerged as important diagnostic targets in so-called “liquid biopsies” [17].

Of note, recently other applications of ExMVs have been considered. For example, they can be loaded with bioactive compounds and drugs as physiological liposomes and used as vehicles for drug delivery [2, 18]. Second, they may be employed in producing anti-tumor vaccines as well as therapeutic agents for infectious diseases and even protection from graft-versus-host disease (GvHD) [2, 19]. Finally, as discussed below, ExMVs are ideal therapeutics to treat tissue and organ injuries, and horizontal transfer of RNA and other molecules here plays an important role.

### ExMVs as signaling devices and vehicles for transfer of cell membrane receptors between cells

The formation of ExMVs was initially described in reticulocytes, and proposed as a mechanism by which maturing reticulocytes employ to shed unnecessary receptors from the cell surface [20]. This observation was followed by the detection of ExMVs released by peripheral blood platelets and other types of cells. In the case of cancer cells, it has been proposed that release of ExMVs allows these cells to shed excess cell membranes [21]. However, as of today, this latter explanation seems to be somewhat trivial.

ExMVs are detectable in all biological fluids, including blood plasma, intercellular fluid, cerebrospinal fluid, urine, sperm, bile, synovial fluid, saliva, and breast milk, as well as malignant effusions and ascites [1–6]. They can be considered as physiological liposomes in which, in the case of ExMVs, a surface phospholipid layer surrounds inner content composed of coding and non-coding RNAs, proteins (e.g., enzymes, signaling components, transcription factors), bioactive lipids (e.g., sphingosine-1-phosphate, prostaglandins, leukotrienes), signaling nucleotides, and metabolites [1–6]. As mentioned above, large ExMVs may also transfer organelles such as mitochondria between cells [22]. Expressed on the surface of ExMVs are receptors, adhesion molecules, and target cell-stimulating ligands—all “hijacked” from parental EXMVs-producing cells. As mentioned above evidence has accumulated that ExMVs may, on the one hand, transfer receptors and cell surface markers between cells [12] and, on the other hand, act as signaling devices by stimulating target cells [10, 11]. These signaling properties and their potential role in cell-to-cell communication were identified almost at the beginning of ExMV research. To support this blood platelet-derived ExMVs were found able to activate endothelial cells [23], polymorphonuclear leukocytes [24], and several leukemic cell lines [11]. The same blood platelet-derived ExMVs also stimulated cytokine secretion and tissue factor (TF) expression in endothelial cells [25], inhibited apoptosis of polymorphonuclear leukocytes [26], and induced chemotaxis of leukemic cells [11]. In addition to platelets, ExMVs are released from normal as well as pathologically altered nucleated cells (e.g., tumor cells) to directly stimulate target cells, and several elegant studies have described these interactions [1–6]. The interaction of target cells with ExMVs may result in increasing proliferation and the inhibition of apoptosis, but in some situations ExMVs may actually inhibit cell growth [27–29]. These direct stimulatory or inhibitory effects depend on the repertoire of signaling molecules expressed on the surface of ExMVs. In support of this notion, we found for example that ExMVs (i) chemoattract human leukemia cells; (ii) increase their adhesion, proliferation, and survival; and (iii) activate various intracellular signaling cascades, including MAPK p42/44, PI3K–AKT, and JAK–STAT [11]. As of today, the signaling role of ExMVs has been reviewed in several excellent and comprehensive review papers [2–6, 30–34].

Besides soluble components such as growth factors, cytokines, chemokines, bioactive lipids, and extracellular nucleotides, ExMVs are part of a broadly understood “cell secretome” that is released from cells into the microenvironment [16]. Currently, much painstaking work has been done to identify proteins, lipids, and RNA components of ExMVs [2]. It is obvious that the composition of the molecular signature of ExMVs varies depending on (i) the cell type that releases these spherical membrane fragments, (ii) the cell cycle status, (iii) the cell activation state, and (iv) the physiological or pathological conditions of the secretome-producing cell.

In addition to playing the role of signaling devices, ExMVs may fuse with cell membranes and thus transfer receptors from the cells of origin to target cells. This has been demonstrated by the transfer of CD41 antigens from blood platelets to hematopoietic cells [12]. This may explain a high expression of CD41 antigen on surface of hematopoietic stem cells (HSCs) that are derived by leucophoresis from mobilized peripheral blood. Namely, during leucophoresis procedure platelets become activated in plastic tubing and release CD41^+^ ExMVs that subsequently may fuse with membranes of HSCs and after fusion render them CD41^+^ [12]. To support this phenomenon transfer of CD41 and certain other platelet-derived receptors (CD61, CD62) to the surface of HSCs may facilitate their engraftment after transplantation [12]. On the other hand, as unwanted effect outdated peripheral blood platelets may be highly enriched for ExMVs that transfer the same molecules (CD41, CD61, CD62), which are involved in platelet adhesion to endothelium, to tumor cells and thus make cancer cells “sticky” to endothelium and predispose them to metastasis [35]. This is one of the reasons why outdated platelets should not be infused into cancer patients. A similar transfer of cell membrane receptors has been observed in many cases and in glioblastomas for example, ExMVs-derived from glioma cells transfer the oncogenic receptor EGFRvIII to neighboring cells and thus increase their transforming potential [36].

Transfer of HIV-entry receptors by ExMVs from monocytes (e.g., CD4 or CCR5) or blood platelets (CXCR4) may render other CD4^−^CXCR4^−^CCR5^−^ cells sensitive to HIV entry [37, 38]. Interestingly, another rare ExMV-mediated possibility exists in HIV infection, as it has been demonstrated that HIV may be carried as cargo inside ExMVs and transmit HIV by ExMV fusion with uninfected target cells. This latter possibility of rare HIV transfer has been described by mythological analogy as a “Trojan horse effect” [39, 40]. A similar mechanism has been postulated for the spread of prion infection [41, 42].

It is also worthwhile to mention that the transfer of receptors by ExMVs released from mature hematopoietic cells during cell processing may create a problem for cleanly isolating lineage-negative hematopoietic stem/progenitor cells (HSPCs) using their cell surface marker expression. Such a procedure during so-called lineage depletion may lead to the loss of the most primitive HSPCs, which after fusion with lineage-positive hematopoietic cell-derived ExMVs are themselves falsely stained as lineage-positive cells [43]. Moreover, it has also been demonstrated that the removal of erythrocytes by cell lysis employing hypotonic solution may lead to generation of erythrocyte-derived ExMVs that highly express annexin V on their surface, which after transfer to target cells (e.g., HSPCs), marks them falsely as cells that have initiated apoptosis [44]. These two examples demonstrate how important is to consider that ExMVs released during cell purification may change cell surface phenotype.

### Horizontal transfer of bioactive molecules between cells by ExMVs

In addition to the role of ExMVs in directly stimulating target cells and their role in transfer of cell surface receptors and markers, horizontal transfer of bioactive molecule cargo present inside ExMVs is their most important biological effect. However, as is common in science, novel observations require time to be accepted by the scientific community. It took us almost 3 years to publish our original work on horizontal transfer of functional mRNA and proteins between cells by ExMVs after our initial report presented at the American Society of Hematology Meeting 2003 in San Diego [45]. Our original paper, which was rejected from some top journals due to disbelief in the results, was finally accepted for publication by one of the less-conservative journal and appeared online by the end of 2005 [13] and as for today is cited several hundred times.

In this aforementioned paper we assumed that the maintenance of pluripotency and the undifferentiated propagation of embryonic stem cells (ESC) in in vitro cultures both require tight cell-to-cell contacts and effective intercellular signaling, and we have hypothesized that ESC-derived ExMVs express stem cell-specific molecules that support self-renewal and expansion of normal adult stem cells [13]. We found that, in fact, ESC-derived ExMVs enhanced survival and improved expansion of murine HSPCs and upregulated the expression of early pluripotent stem cell (October 4, Nanog, and Rex-1) and early hematopoietic stem cell (Scl, HoxB4, and GATA 2) markers in these cells. Furthermore, molecular analysis revealed that ExMVs from ESCs express Wnt-3 protein and are selectively highly enriched in mRNAs for several pluripotent transcription factors compared with parental ESCs. What is even more important, as demonstrated in that work these mRNAs could be horizontally transferred to HSPCs and translated into the corresponding proteins [13]. This biological effect was inhibited after heat inactivation or pretreatment with RNAse, indicating a major involvement of protein and mRNA components in the observed phenomena [13].

Following this report, other very elegant studies confirmed the presence of horizontal transfer of mRNA via ExMVs derived from models of glioblastoma [15], murine and human mast cells [14], lung cells [46], endothelial cells [47], and mesenchymal stem cells [48]. Specifically, glioblastoma-derived ExMVs were found to be enriched for mRNA, miRNA, and proangiopoietic proteins and, after horizontal transfer of these molecules, promoted angiogenesis in growing tumors and stimulated tumor cell proliferation [15]. In another elegant work, ExMVs were demonstrated by microarray analysis to contain mRNAs for 1300 genes, and after ExMV-mediated transfer of murine mRNA to human cells, murine-derived proteins were found to be expressed in recipient human cells [14]. Similar results were obtained in another very elegant study performed by another group of investigators in which lung cell-derived ExMVs transferred translationally active mRNAs into human hematopoietic cells, which led to the expression of lung-specific proteins in  these cells [46, 49]. Finally, the phenomenon of horizontal transfer of specific mRNA subsets carried by endothelial cell-derived ExMVs to human umbilical vein endothelial cells (HUVECs) has been convincingly shown in another elegant work [47]. This ExMV-mediated transfer promoted the induction of angiogenic potential in HUVECs in in vitro cultures. A similar effect has also been observed in vivo in SCID mice when human mammary epithelial cells (HMECs) exposed to endothelial cells-derived ExMVs were embedded in matrigel implants and transplanted subcutaneously to these mice [47].

In the following years, as follow up the phenomenon of horizontal transfer of RNA was demonstrated for ExMVs derived from peripheral blood platelets, hematopoietic CD34^+^ cells [50, 51], and CD133^+^ cells [52]. It has also been shown that this novel mechanism occurs also for noncoding RNA and miRNA [5, 14, 49, 52]. Thus, ExMVs enriched in RNA species became a focus of interest in several biological processes related to tissue and organ regeneration and as a potential diagnostic tool to detect pathological RNA species in the abovementioned liquid biopsies performed on samples aspirated from biological fluids (e.g., from blood plasma, ascites, pleural effusion) [17].

Importantly, it has been demonstrated in several elegant studies that ExMVs derived from cells employed in regenerative medicine in therapy (e.g., mesenchymal stromal cells) may also transfer several mRNA and miRNA species in a horizontal manner [53]. After delivery to the target cells, these are translated into proteins or regulate the expression of genes that inhibit apoptosis and/or promote angiogenesis in damaged tissues, such as kidney, myocardium, or liver [2, 16].

So far, horizontal transfer of RNA by ExMVs has been demonstrated for many types of normal and transformed cells and is also reportedly not species specific. It may be particularly important in the cell-to-cell communication mechanism employed by stem cells, which lack gap junctions, in interacting with other cells in the microenvironment [1–6, 13, 54]. It would be worthwhile to better explore how this mechanism is involved in the crosstalk of stem cells with other cells that comprise their specific niches.

Interestingly, it has been recently reported that a novel type of intestinal cells, known as a telocyte, secretes ExMVs to exert its regulatory functions in the tissues [55]. Telocytes may affect the biology of several cell types, including differentiated somatic cells and tissue-residing stem cells [56]. Evidence has accumulated that ExMVs are mediators of several long-distance paracrine functions of the telocytes residing in adult organs. Further work is needed to elucidate how these interactions are regulated via paracrine telocyte-mediated signals, including the release of ExMVs. As reported, telocytes express several miRNAs with pro-angiopoietic potential (miR-126, miR-130, let-7e, and miR-100), and their number increases in the myocardium after heart infarct [57]. Therefore, the potential horizontal transfer of these miRNA species to the damaged myocardium via ExMVsmost likely promotes angiogenesis [58].

There are several other excellent examples of the role of ExMVs in horizontal transfer of RNA and other bioactive molecules, but, because of space limitations, they necessarily exceed the scope of this review. We therefore apologize to the various authors for not discussing several of these examples.

### Novel potential applications of ExMV-based strategies by utilization of horizontal transfer of RNA and other molecules

**Fig. 2:**
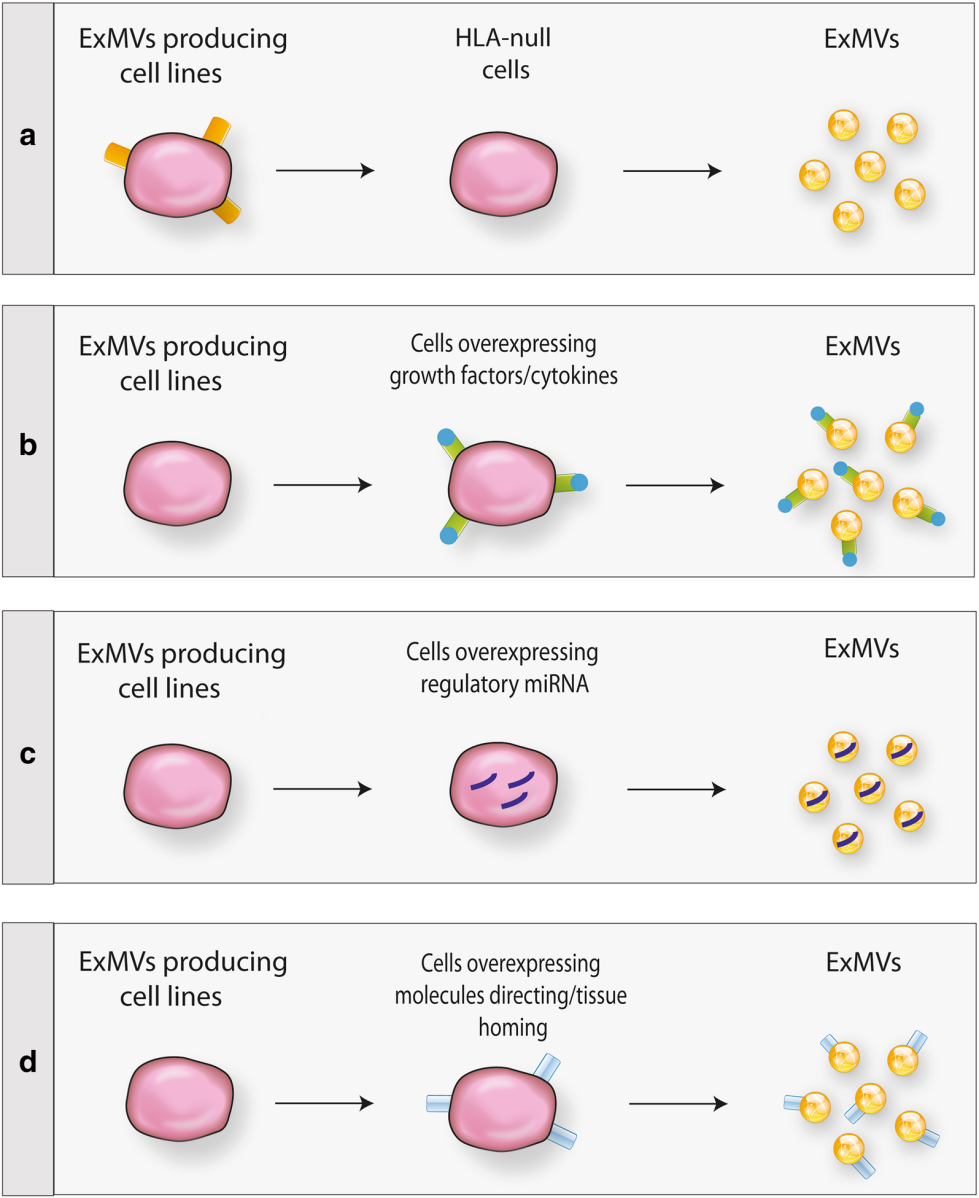
Different possible approaches to generating more efficient pro-regenerative ExMVs. ExMVs could be harvested from large-scale in vitro cultures of producing cell lines—for example, mesenchymal stem cells or induced pluripotent stem cells. Such cell lines may be modified to obtain ExMVs that do not express HLA antigens (**a**), are enriched in growth factors, cytokines, chemokines and bioactive lipids that promote regeneration of damaged organs (**b**), are enriched in mRNA and regulatory miRNA facilitating regeneration of damaged tissues and/or promoting angiogenesis (**c**), or display on their surface molecules that direct them to, and cause them to be retained in, damaged tissues (**d**) (adapted from Ratajczak et al. [16])

As mentioned above, it has been very convincingly shown in seminal work that ExMVs derived from mesenchymal stromal cells exert similar biological effects in models of tissue and organ regeneration in vivo as intact mesenchymal cells [53, 59]. This important observation opened new possibilities for therapeutic application of ExMVs, and these seem to be promising candidates for therapy, as they avoid problems related to the application of intact cells [2, 53, 59]. Thus, it is possible to modulate ExMV-producing cells to engineer modified ExMVs to employ them more efficiently for tissue and organ regeneration in vivo based on their paracrine effects, including horizontal transfer of RNA molecules (Fig. 2).

For example, ExMVs could be isolated for potential applications in regenerative medicine from a large-scale ex vivo expansion of cultured cells, for example, mesenchymal stem cells, in an appropriate culturing system. Taking advantage of epigenetic memory in induced pluripotent stem cell (iPSC)-differentiated cells, one can also envision that, for example, ExMVs from iPSCs obtained from dermal fibroblasts or iPSCs differentiated into epidermal cells would preferentially affect the regeneration of damaged skin (e.g., after burns) [16]. Similarly, ExMVs isolated from the supernatants of iPSCs derived directly from cardiac fibroblasts or differentiated toward cardiomyocytes would have advantages in the regeneration of damaged myocardium. This strategy, which we proposed in the past [16], has been recently successfully employed and reported in a recent paper [60].

In addition, as shown in Fig. 2, ExMVs-generating cells could be genetically modified in order to produce custom-engineered ExMVs more suitable for therapy [16, 61]. First, as depicted in Fig. 2a, if it would be possible to generate and subsequently expand ExMV-producing cells that lack genes encoding histocompatibility antigens. This approach would minimize the possibility of cross-immunization with donor HLA antigens. Second, ExMVs-producing cell lines could be transduced with genes that overexpress on the cell surface (i) peptides that protect target cells in damaged organs from apoptosis and stimulate proliferation of the residual remaining cell population (e.g., stem cell factor or Notch ligands) or (ii) factors that effectively induce angiogenesis (e.g., VEGF, FGF-2, or SDF-1) (Fig. 2b). As a third possibility, ExMVs-producing cell lines could be enriched for mRNA and regulatory miRNA species that, after horizontal transfer to the damaged tissues, promote regeneration by inhibiting apoptosis and promoting angiogenesis (Fig. 2c). For example, we speculate that ExMVs derived from producer cell lines cultured in hypoxic conditions would be enriched in mRNAs and miRNAs that promote angiogenesis. Finally, we envision that ExMVs-producing cell lines could be enriched for molecules that facilitate their tropism to the damaged organ and subsequently promote retention of ExMVs in the damaged tissues (Fig. 2d).

## Conclusions

Solid evidence has accumulated that ExMVs can transfer mRNA, miRNA, and large non-coding RNA molecules in addition to proteins, bioactive lipids, metabolites, and signaling nucleotides between cells in a horizontal manner [13–15, 46, 48, 52, 62, 63]. This phenomenon is involved in several physiological (e.g., tissue and organ regeneration, angiogenesis) as well as pathological (e.g., cancerogenesis) processes. Further work is needed to better purify ExMVs, separate them from apoptotic bodies, and identify the full pattern of bioactive molecules, including all RNA species, that are present in ExMVs derived from different cell types. It will be important to decipher the molecular signature of mRNAs in ExMVs circulating in normal blood in human individuals, depending on sex and age.

Also important, there are emerging possibilities for more efficiently utilizing ExMVs in tissue and organ regeneration by generating ExMVs enriched in bioactive molecules, including the optimal signature of mRNAs and miRNAs for inhibiting cell apoptosis and increasing angiogenesis of damaged tissues [16, 61]. Finally, more work is needed to shed more light on the secretory mechanism involved in ExMVs release from cells and their shedding from the cell surface membranes. Also needed are more efficient large-scale ExMVs isolation and purification methods.
